# Iran Quality of Care in Medicine Program, a Gateway to Collecting Healthcare Data in the Absence of Nationwide Digitized Health Information

**DOI:** 10.34172/aim.2023.20

**Published:** 2023-03-01

**Authors:** Reza Malekzadeh

**Affiliations:** ^1^Professor of Medicine Digestive Disease Research Institute, Shariati Hospital, Tehran University of Medical Sciences, Tehran, Iran

 Data and information are essential for healthcare system stewardship. A population-representative healthcare dataset, analytics tools, and management techniques assist in preventing, intervening, and managing health problems. Among all the required data, gathering national and subnational data on healthcare utilization, expenditures, and quality is one of the critical first steps to improving patients’ experience of the disease and the healthcare system. In countries with well-established economies such as the US, where a functioning healthcare data infrastructure exists, healthcare providers enter data relevant to the patient’s experience into a digital platform in an almost real-time fashion. Health insurance claims data, for instance, are generated at the point of care in the US. An insurance claim includes several variables, such as the type and timing of the visit, the pharmacy used, the condition, comorbidities, and the cost information. In addition, electronic health records improve the quality and quantity of the data by adding more clinical information to the existing claims. This enrichment primarily results from adding clinical lab test results, medication information, and imaging data. Despite several attempts to digitize patient visits at the point of care for over two decades, most healthcare systems are still analog and balkanized in Iran. Developing an integrated digital healthcare infrastructure in developing countries, including Iran, has shown some promise, but these capabilities still need to be supplied.^1^ Due to the rapid health transition, chronic conditions have replaced infectious diseases as the primary driver of utilization and healthcare costs. Developing countries need more accurate and relevant population data for a small number of chronic diseases that cause most of this healthcare burden.^2^

 In this issue of the Archives of Iranian Medicine, Shahraz et al published the study protocol of the first attempt to generate data on the national patients’ experience in Iran.^3^ IQCAMP was a demonstration project launched in 2016 to examine a survey method that could fill the healthcare data gap for high-burden conditions. Diabetes, congestive heart failure, acute myocardial infarction, chronic obstructive pulmonary disease, end-stage renal disease, stroke, and major depression are typical conditions that consume the most healthcare resources in the 21st century. The authors used an ingenious sampling technique^4^ to represent the nation. However, the small sample size used in this demonstration study made subgroup analysis and statistical inference challenging.

 Nevertheless, the study gave the research and policymaking communities hope that periodic nationwide sampling could monitor the quality and cost of high-burden conditions. From the beginning, the study used a participatory approach. Clinical guidelines and quality indicators were adapted to fit current healthcare practices without compromising scientific definitions. The study team made regular monthly contact with local medical staff and physicians for three months. Trained nurses recruited patients at the point of care for all conditions except diabetes. The diabetes survey participants were randomly selected from a national study. The nurses interviewed the patients four times to obtain baseline data and three monthly follow-up interviews. They did not intervene with the care given to the patients at the time. There has been a relatively high response rate of nearly 80%, a slight loss to follow-up, and a 66% completion rate of all four planned visits.

 The study was completed involving 156 physicians, 78 nurses, 23 members of the disease-specific expert committee, and 11 data and research specialists, including engineers who contributed to building the digital platform for data entry on provisioned devices. This effort was the first in Iran to gather national-level data on the cost and quality of healthcare services for these seven high-burden diseases. The collected data are invaluable as they quantify the country’s healthcare quality. According to the authors, these studies should be integrated into disease-specific national registries or conducted periodically as healthcare surveys.
